# The basics of good postoperative care after glaucoma surgery

**Published:** 2016

**Authors:** Fatima Kyari, Mohammed M Abdull

**Affiliations:** Ophthalmologist: Department of Ophthalmology, College of Health Sciences, University of Abuja, Nigeria.; Ophthalmologist: Ophthalmology Department, Abubakar Tafawa Balewa University Teaching Hospital, Bauchi, Nigeria.

**Figure:**
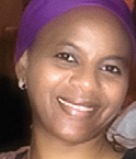
Fatima Kyari

**Figure:**
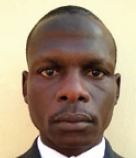
Mohammed M Abdull

Glaucoma patients are treated by lowering the intraocular pressure (IOP) to a level that it is not harmful to the optic nerve. This prevents or delays loss of vision. Lowering of the IOP can be achieved through use of eye medication, surgery or laser procedures. The most common glaucoma surgery is trabeculectomy. This entails creating an additional passage for the drainage of the fluid inside the eye (the aqueous humour). The fluid drains from the anterior chamber, through an opening (fistula) in the sciera, to an artificially created reservoir (the bleb) under the conjunctiva. The bleb enables the fluid to be absorbed gradually into the systemic circulation and is hidden under the eyelid.

Before patients have a trabulectomy, they must be informed that the operation will not cure the disease; it will lower the IOP in order to reduce the rate of deterioration of vision loss. They must understand that any vision already lost cannot be regained through surgery and that the surgery may cause initial blurring of vision in the immediate postoperative period (and will resolve by itself overtime).

Good follow-up care is essential, and patients should be provided with a contact number to call when they need to complain, ask for information or reschedule an appointment, or when they notice any symptoms that could indicate a complication.

Postoperative care after trabeculectomy can be classified into immediate postoperative care (0-6 weeks) and mid- to longer-term postoperative care (after 6 weeks).

## Principles of immediate postoperative care (0–6 weeks)

### Ensure that the aim of surgery has been achieved, i.e that the IOP has been lowered

One day after the operation (on day 1), the surgeon examines the eye to ensure that the operation is achieving drainage of aqueous humour with adequate formation of a bleb and satisfactory lowering of the IOP The IOP on the first day postoperatively is not the final IOP but serves as a good indication that a drainage channel has been successfully created. The surgeon also examines the eye to look for early complications at this stage: infection, hyphaema, conjunctival/wound leak, shallow/flat anterior chamber, hypotony requiring intervention, and choroidal detachment.

**Figure:**
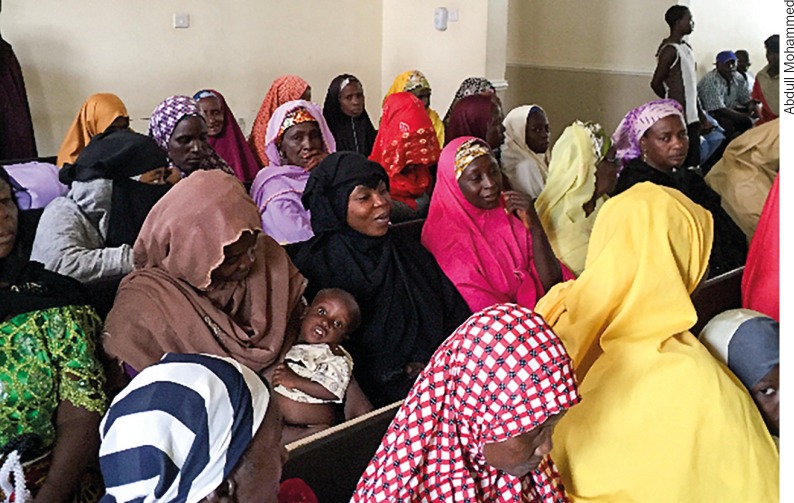
Patients wait for their follow-up examination, which will include IOP measurement – an essential component of postoperative glaucoma care. NIGERIA

### Protect the eye from external injury

The operated eye is padded until the following day. If the other eye has no vision, the operated eye is not covered but a perforated eye shield is placed on it instead.

### Ensure hygiene and prevent infection

The patient should keep the face clean and avoid touching the eye. Patients may bathe and shower, taking extra care not to bend forward orto touch the operated eye (which may also be protected with an eye shield). Hands should be washed before instilling any eye drops. Postoperative antibiotic eye drops (e.g. chloramphenicol) are prescribed for use 4–6 hourly or 4–6 times a day for 2–3 months.

### Reduce inflammation associated with the operation

Some degree of redness and swelling may occur after the operation. Postoperative anti-inflammatory eye drops (e.g. dexamethasone) are prescribed for use 1–2 hourly during the first few days and subsequently reduced to 4–6 times a day. The postoperative eye drops may be used for 2–3 months as advised by the reviewing doctor.

### Control pain

It is usual to have some eye pain after glaucoma surgery but this is often mild and responds to analgesics such as non-steroidal anti-inflammatory drugs and acetaminophen.

## Symptoms and signs of complications (0–6 weeks)

### A sudden loss of vision

A small reduction in vision, usually not more than 2 lines of visual acuity (VA), may occur after surgery, but should improve gradually or at least not worsen rapidly. Rapid deterioration of vision is an emergency; therefore it must be reported promptly. The following are common causes.

1. **A hyphaema** indicates the presence of blood in the anterior chamber. This clogs the trabecular meshwork and blocks the fistula created for drainage to the sub-conjunctival space, causing the IOP to rise, sometimes catastrophically. This increases damage to an already diseased optic nerve and may result in blindness if not promptly reported and treated. Patients should report to the health facility where they had the surgery for urgent management.
  **Sudden loss of central vision** may occur, especially in patients who had very severe disease at the time of surgery. Surgeons sometimes make a decision to avoid operating on such patients but instead offer other, less invasive, alternatives. Vision loss may be gradual or rapid, depending on the severity of disease and postoperative inflammation.
2. **Choroidal detachment** is caused by the passage of serum into the supra-choroidal space (between the sciera and the choroid) due to increased transmural pressure, most frequently caused by globe hypotony following trabeculectomy. It can present with quite severe loss of vision with variable degrees of pain. An urgent B-scan ultrasound can help with the diagnosis. Urgent treatment is needed to prevent permanent loss of vision.

**Figure:**
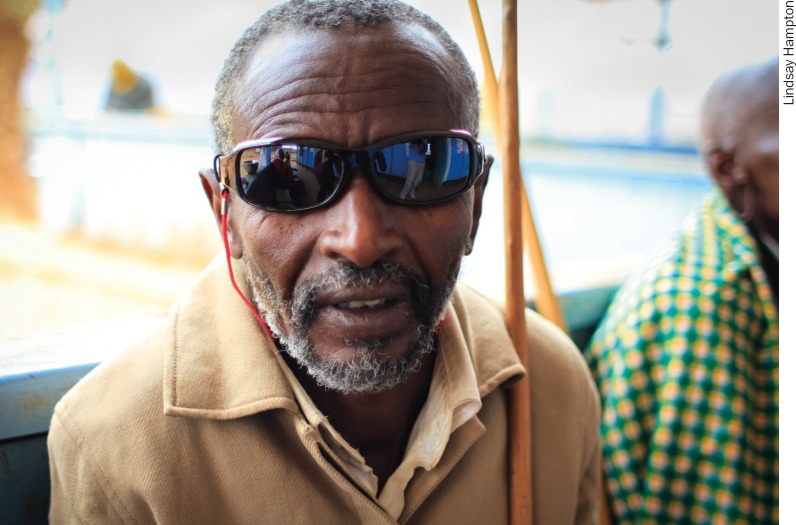
After a trabeculectomy, eyes require special care and protection from injury, e.g. wearing UVB sunglasses during the daytime. KENYA

### Soft eye

This leads to a shallow or flat anterior chamber. It is usually caused by over-filtration due to a loose scierai trabeculectomy flap, a conjunctival wound leak at the incision site, or a leak via a conjunctival buttonhole. It mayor may not present with reduction in vision, with little to severe pain depending on the cause. Padding the eye may be sufficient, but urgent surgery is sometimes necessary.

### Redness, pain and discharge (pus)

This may be accompanied by a possible drop in VA very soon after surgery, and this combination is usually indicative of an active infection. Redness alone may be normal following surgery but if it persists beyond a few days it should be reported as it may mean an active inflammation in the eye. All instances of the above symptoms should be reported urgently to the health facility where they will be investigated and properly treated.

## Principles of longer-term postoperative care (after 6 weeks)

### Optimise vision

Six to eight weeks after the operation, refraction should be undertaken to assess the patient's best-corrected visual acuity (BCVA) and to obtain a prescription for spectacle or contact lens correction. Not everyone can wear/continue with contact lenses following trabeculectomy. The doctor must assess the bleb and the suitability of contact lens wear.

### Continue to protect the eye

Advise the patient about protecting their eye. Especially in sports, physical contact activity and windy weather, the eye needs to be protected from injury with sports goggles (where indicated) or UVB sunglasses during outdoor activities such as riding a motorcycle. The protective eyewear should be kept clean.

### Continue medication

When necessary, the postoperative medication (antibiotics and steroid eye drops) may be continued for up to 3 months after surgery on advice of the doctor.

In some cases, anti-glaucoma medication may also be prescribed after the operation, if the lowering of the IOP to the desired level has not been achieved. Patients should be made aware of this possibility before surgery.

### Be alert for signs of postoperative complications

The patient must be monitored regularly to detect any changes in vision, pain or any other symptoms that will indicate postoperative complications such as infection, a failed bleb or over filtration. The importance of community-based follow-up by the community health worker or ophthalmic nurse cannot be overemphasised; this is essential in order to ensure that symptoms and signs are recognised and treatment offered without delay. Patients should be advised to get help if they notice any symptoms-see panel below.

**‘The importance of community-based follow-up by the community health worker or ophthalmic nurse cannot be overemphasised’**

### Possibility of additional surgical procedures

When the IOP control is not at the desired level, the doctor may advise additional procedures to optimise IOP control. These procedures may include the release of releasable sutures, bleb revision, antimetabolite injections or even laser procedures.

## Symptoms and signs of complications in the longer term (after 6 weeks)

1. **Redness associated with discharge (pus) from the eye.** Long after a successful trabeculectomy, bacterial infection could occur. A person who has had eye surgery and has discharge (pus) from the eye needs to be seen immediately by an eye doctor and treated with the appropriate medication. Self-medication, especially with steroid eye drops, must be avoided. Serious consequences and loss of vision could occur if there is endophthalmitis.
2. **Discomfort.** A large drainage bleb may cause abnormalities in tear spread over the cornea, causing poor tear films that cause a sensation of dryness and discomfort. Such large blebs may also be uncomfortable under the eyelid causing cosmetic embarrassment.
3. **Cloudy vision and cataract.** The chance of an eye developing a cataract increases after trabeculectomy. The patient should be made aware of this. The patient may have increasing glare in bright sunlight or while driving at night. Any reduction in vision must be investigated to determine the immediate cause. Vision generally improves following cataract surgery, except if the glaucoma damage is significant.
4. **Changes in refraction:** There may be astigmatism following trabeculectomy because of the mild distortion of the eye's anatomy. This may manifest as a need for new spectacles. Such change can be delayed until about 3 months after the operation, when the eye has stabilised.
5. **Continued loss of vision:** Even with good IOP control, patients may still continue to lose vision. The patient may see haloes around light bulbs which may indicate cloudiness of the cornea due to raised IOP Glaucoma surgery reduces the rate of loss of vision in glaucoma patients but may not completely halt it.

**Advice for patients at discharge**

Patients should be given information about the following before they go home after a trabulectomy. They should also understand about the possible complications and understand the importance of getting help urgently so that their vision can be preserved. Make sure that patients have the contact information they need, e.g. the telephone numbers of the appropriate person so that they can get an appointment as soon as possible.

**How the eye will feel**

You may have some watering, sandy sensation or blurring of vision after trabeculectomy, but this should clear within a few days. Soreness and irritation may occur from the sutures or because of the surgery itself. These sensations generally reduce within a few days.

**Protection**

The eye has now been operated on and is more fragile than before. It is important to take special care and to protect your eye from injury. You can wear UVB sunglasses in the daytime.

**Caution with activity**

Physical activities that require bending forward such as farming, ‘ruku’ and ‘sajda’ (prostration) during Muslim prayer and lifting of heavy items are to be avoided in the first six weeks after surgery. Strenuous activities such as running, jumping, swimming and sex are also to be avoided until the eye doctor advises it is safe to resume them.

**Cleanliness and hygiene**

- You can shower, have a bath or wash your face to ensure cleanliness.
- For at least one week, do not use eye make-up, including kohl and eye pencil.
- Avoid touching the eye directly or rubbing it.

**Medication**

- Wash hands before applying your eye drops.
- Do not touch the tip of the dropper of the eye drop bottle with fingers and do not allow the tip of the bottle to touch the eye
- Use the eye drops as often as indicated on the bottle or as directed by your doctor.

**Keeping appointments**

It is important to keep your appointment, as the eye doctor will need to regularly monitor your vision and eye pressure and look out for any signs of complications. Bring your eye drops with you to the hospital.

**IMPORTANT: Come back in case of any worrying signs or symptoms**

Contact your community health worker (if you have one) or your eye nurse or eye doctor immediately if you experience any of the signs or symptoms listed below-even if this is several months after the operation – as these can indicate that there is a problem that needs to be looked at. Coming back quickly will give medical professionals the best chance to save your sight and your eye.

- Any pain: come back very urgently
- A rapid reduction in vision (particularly central vision): come back very urgently
- Redness and/or discharge (pus): come back very urgently
- Haloes around light bulbs: come back very urgently
- Blurry or distorted vision (including increased glare in sunlight or while driving at night): less urgent, but can easily be corrected with a cataract operation or a new spectacle prescription.

